# Delphi Study on the Contextualization of Recommendations for Promoting Healthy Eating in Urban Settings of Latin America and the Caribbean

**DOI:** 10.3390/nu16234017

**Published:** 2024-11-24

**Authors:** Nayara Tamayo-Fonseca, Elisa Chilet-Rosell, Marta Puig-García, Gregorio Montalvo-Villacis, María Fernanda Rivadeneira, María Jose Sanchis, Working Group of Public Policies to Promote Healthy Diets in Urban Contexts in Latin America and the Caribbean, Lucy Anne Parker

**Affiliations:** 1 Research Unit for the Analysis of Mortality and Health Statistics, Community Health Research Group, Department of Community Nursing, Preventive Medicine, Public Health and History of Science, University of Alicante, 03690 Alicante, Spain; 2 Department of Public Health, Universidad Miguel Hernández de Elche, 03550 Alicante, Spain; echilet@umh.es (E.C.-R.); marta.puigg@umh.es (M.P.-G.); msanchis@umh.es (M.J.S.); lparker@umh.es (L.A.P.); 3 CIBER de Epidemiología y Salud Pública (CIBERESP), 28029 Madrid, Spain; 4 School of Medical Specialities, Colegio de Ciencias de la Salud, Universidad San Francisco de Quito, Quito 170901, Ecuador; gregoriomontalvovillacis@gmail.com; 5 Institute of Public Health, Faculty of Medicine, Pontificia Universidad Católica del Ecuador, Quito 170143, Ecuador; mfrivadeneirag@puce.edu.ec

**Keywords:** noncommunicable diseases, Latin America and the Caribbean, health promotion, healthy public policies, Delphi process

## Abstract

**Background.** International public health agencies recommend policies to improve diets and promote healthy eating, but implementation often falters due to varying contextual factors across regions. **Objectives.** This study evaluates the relevance and applicability of these policies in urban areas of Latin America and the Caribbean (LAC). **Methods.** Using the Delphi technique, we convened a panel of 21 experts from 13 LAC countries, representing public policy, research, social action, and healthcare. Over two consultation rounds, the panel assessed 21 potential actions that local actors could implement to promote healthy eating by altering the physical and social environments. Data analysis led to a consensus on classifying these actions as high priority, low priority, or debatable. **Results.** The panel highlighted several contextual factors affecting policy implementation in the LAC region. For example, the informal nature (such as informal street vending) of many food establishments in the LAC region complicates zoning policies, such as restrictions in areas near schools, making them difficult to enforce and likely to face resistance. **Conclusions.** The panel identified eight actions as high priority, eight as low priority, and five as debatable for implementation at local level in the LAC region.

## 1. Introduction

Noncommunicable diseases (NCDs) and their risk factors are a leading cause of morbidity, mortality, and disability worldwide [1]. NCDs cause an estimated 41 million deaths annually, of which 15 million are premature [2]. In the Latin American and Caribbean (LAC) region, there were an estimated 5.5 million NCD-induced deaths in 2016 (81% of total deaths), of which almost 40% were premature, occurring before 70 years of age [3].

Obesity is a major public health problem both in high-income countries and in low- and middle-income countries [4]. In the LAC region, obesity rates have increased substantially over the last 20 years [5,6]. Some estimates show that in 2022 there were 4.2 million (8.6%) overweight children under 5 years of age in LAC [7], exceeding the global average of 3.9 million (5.6%). This increase affects not only minors, but also children of all ages, adolescents, and women in all countries of the region, being more pronounced in urban areas, and particularly among the most impoverished groups [8,9].

Obesity is considered a risk factor for diet-related NCDs such as cardiovascular disease, certain types of cancer, and diabetes [10], all of which have major implications for individuals, families, societies, and economies. In some countries, the epidemic of overweight and obesity coexists with undernutrition and micronutrient deficiencies, resulting in a “double burden” of malnutrition. As mentioned above, 8.6% of children were overweight, but, also, 7.4% of people lived in hunger; in addition, one in three inhabitants of LAC countries did not have access to nutritious or sufficient food, due to a lack of economic or other resources [8]. In this context, many countries are facing a complex epidemiological and nutritional transition [11,12,13], with overlapping burdens of malnutrition and interrelated factors that determine diet quality and eating habits impacting on nutrition and health [8,13]. The impact of the double burden of malnutrition is high in the LAC region and continues to be considered one of the greatest public health challenges in LAC countries, as it affects the physical and cognitive development of vulnerable populations as well as increases the risk of NCDs among the general population. Case studies conducted in eight LAC countries showed that the double burden is a problem for the health of the population, but also slows down the development of countries, generating enormous losses in productivity and higher costs in health systems [14].

To alleviate these detrimental effects, both on the quality of life of individuals and on the social and economic development of the affected countries, joint actions are needed in the areas of policies, health systems, and communities [15].

In addition, the complex landscape in LAC is marked by rapid urbanization, with growing cities and substantial distances between urban areas and food production sites. Coupled with the expansion of ultraprocessed food industries, supermarket growth, and fast-food proliferation, these challenges are further compounded by issues of gender inequality, income disparities, poverty, social vulnerability, health disparities by ethnicity, displacement, political insecurity, varying levels of social support, and community organization, as well as post-pandemic economic instability [7,14,16,17,18]. This scenario underscores the urgent need for the implementation of contextually tailored and effective policies to promote healthy diets and mitigate the negative impacts on urban health.

Public health institutions and national governments in many regions of the world have developed effective strategies for preventing and controlling NCDs and their risk factors [1,19,20]. However, various organizations have warned that the proposed reduction targets are unrealistic, including those related to malnutrition [2,15]. They recognize the need for urgent action to address the complex problems caused by the increasing burden of NCDs [9]. For this reason, various international bodies and organizations have issued guidelines and recommendations for implementing healthy public policies. For example, the World Health Organization recommends a combination of fiscal, legislative, and environmental measures to promote healthy eating [1]. Other institutions have developed specific guidelines related to food environments, so countries can evaluate progress on policies [21] and monitor various components related to food systems [22].

Despite the extensive evidence and recommendations currently available, the implementation of policies to reduce obesity and NCDs is poor [10]. Most of the evidence for improving diets is generated in only a few countries [4], and the limited knowledge and adaptation of relevant recommendations could compromise policy implementation in low-resource settings [23]. It is, therefore, necessary to understand the factors that are hindering the implementation of actions and to adapt the evidence to improve the implementation of policies at the local level in these settings.

In view of the scarce information on the contextualization of actions in urban environments in low-income settings, we aimed to analyze the perceived contextual relevance and applicability of local actions recommended by international public health agencies for promoting healthy eating in urban areas of LAC. We employed the Delphi method to mine local knowledge and achieve consensus opinions.

## 2. Materials and Methods

**Figure 1:**
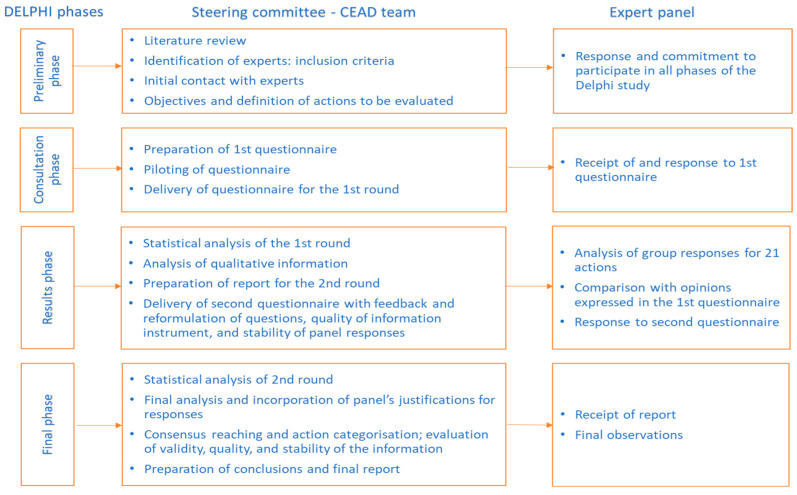
Schematic process of the main study phases.

To meet our study objective, we planned and organized a Delphi study in several phases, as shown in Figure 1.

### 2.1. Local Policy Actions to Promote Healthy Eating

We obtained the policy items or actions by mapping the evidence-based policy measures proposed by public health institutes or organizations for promoting healthy eating through modification of the local social and/or physical environment [24]. We were interested in concrete actions (e.g., introducing a tax on sugary drinks) rather than general objectives (e.g., discouraging the consumption of sugary drinks). The first step of the mapping process was identifying the government-supported public health institutes or organizations that generate evidence-based recommendations on policy actions (National Institute for Health and Care Excellence (NICE) in the UK, Community Preventive Services Task Force (CPSTF) in the USA, and WHO and its regional offices). The second step was identifying other public health institutes through the International Association of National Public Health Institutes (IANPHI; 115 members in 98 countries). Next, we extracted the policy measures proposed by each institute. After reviewing all the extracted information and identifying similarities and differences, we synthesized the recommendations in a list of actions that could be implemented at the local level. In total, we analyzed 21 evidence-based policy actions focused on modifying the physical and social environment to promote healthy eating at the local level.

### 2.2. Participants

We assembled a panel of experts with proven expertise in public health, policy, health systems, health promotion, disease prevention, food sovereignty, and healthy eating in urban settings across LAC countries. Experts were selected based on predetermined criteria, including competence, experience, availability, and impartiality, ensuring a minimum of 15 participants per round, gender equity (predetermined as at least a 60:40 split; finally 13 women and 8 men participated), and representation from at least 8 LAC countries (finally, individuals from 13 LAC countries participated), with no more than 3 individuals from the same country (this criterion was not fully met due to overrepresentation from Ecuador). The panel comprised experts from political, academic, community, and healthcare sectors. Recruitment was conducted through academic searches (health databases, technical reports, and gray literature on collective, social, and political actions), snowball sampling, and personal contacts, with clear communication about study objectives, timelines, and participation requirements. Multiple reminders were sent to confirm involvement, and those unresponsive after two reminders were excluded. We initially identified 50 experts who met these criteria, of whom 21 participated in the first round (see Appendix B, Table A1 for detailed information about the recruitment process).

### 2.3. Collection of Information

The experts completed an online questionnaire (through Google Forms) that reported their experience in different fields of expertise and geographic level, as well as their assessment of the degree of the applicability and contextual relevance of the 21 policy items or actions. *Contextual relevance* was defined as the importance of the recommendation/action for improving people’s diets, considering the local eating habits of the urban population in LAC, and *Applicability* was defined as the likelihood of a recommendation being successfully developed or implemented in the urban LAC context, where appropriate, e.g., a recommended action would be to use the nutritional traffic light on food packaging. Although its implementation (or applicability) could be high, it may not be the most relevant action in places where foods are prepared and distributed in communities without packaging (e.g., abundance of street venders selling foods with low nutritional value). The experts rated these two dimensions using a 5-point Likert scale, where 1 represented very low applicability/contextual relevance and 5 represented very high applicability/contextual relevance. All questions had a free-text box where participants could explain and justify their scoring decisions and make comments as to whether the wording of the item needed to be clarified. Before the first round, we piloted, corrected, and validated the initial questionnaire. In the second round, we modified the wording of some items based on the information collected in the first round. The panel evaluated the items a second time, explaining their reasoning for any changes in opinion. We also included a contextual prioritization instrument where each member of the panel selected what they considered to be the 5 most applicable and most relevant recommendations in their local context. We included a set of questions regarding the participant’s perceived quality of the information generated and effectiveness of the method Delphi.

### 2.4. Analysis of Information

For each round, we analyzed the qualitative and quantitative information generated by the panel on applicability and contextual relevance.

In the first round, we processed the quantitative data using an adaptation of the methodology described by Landeta J [25]. This involved calculating descriptive quantitative measures (mean, median, standard deviation, coefficient of variation, and quartiles) of the values assigned by the experts on each item of the scale. We created frequency and contingency tables to evaluate interexpert agreement and divergences between the dimensions (applicability versus contextual relevance) of each recommendation. We followed the same process to analyze the quantitative data collected in the second round, and we also evaluated positioning subgroups of items and experts. We calculated the differences in the measures between the two rounds for each dimension.

To process the qualitative data, we evaluated regularities and extremes expressed by the panel for each item [26], and two researchers independently read all the comments and grouped the ideas using open codes. We compared the main ideas and created a list of statements that summarized the participants’ comments in the following three categories: (a) Problems with or comments on wording; (b) Comments on contextual relevance; and (c) Comments on applicability.

We sent a summary of the information in a neutral, nonpositioned way to allow for feedback and subsequent assessment by the panel, including the position of the items in the ranking; the distribution of the group’s score (median with its interquartile range—IQR); the expert’s position in the previous round and a consolidated report of the responses provided by the panel members in the free-text boxes.

In the second round, we analyzed participants’ reasoning for any changes in opinion from the first round and the impact of modifying the wording of the recommendations.

#### 2.4.1. Estimating the Degree of Consensus

Consensus is described as the degree of convergence of individual estimates that is achieved when opinions have an acceptable degree of proximity. We determined consensus as a desirable goal, but this was not forced as a criterion for completion the study.

We constructed a set of criteria to analyze the information and measure the stability of the estimates between the two rounds:

1. To rank the items within the list, we ordered the scores of each item according to mean, median, and standard deviation for each dimension in each round. We created a heat map to analyze the stability of the responses.
2. To estimate the degree of convergence of opinions, we analyzed the degree of clustering of the responses in one of two groups according to the scale: 4/5 (high applicability/contextual relevance) and 1/2 (low applicability/contextual relevance). We considered that an item had good convergence when at least 70% of participants had scored the item in one of these two groups.
3. To estimate the degree of proximity, we analyzed the variation of the standard deviation and coefficient of variation between first and second round for the two dimensions. We considered lower dispersion as indicative of a more robust group response. Finally, to assess contextual prioritization, we counted the number of times the item was voted as the main recommendation in the LAC context.

#### 2.4.2. Categorization of Actions

After completing the steps listed above, we triangulated the information to divide the items into three categories:

1. High-priority policy actions: Actions with the highest mean scores, in positions 1 to 10 in the rankings of applicability and contextual relevance, with a high degree of convergence (>70% of participants selecting 4 or 5 on the Likert scale for both dimensions), a high number of votes in the contextual prioritization instrument, and favorable comments from experts.
2. Low-priority policy action: Actions with the lowest mean scores, in positions 11 to 21 in the rankings of applicability and contextual relevance, with a high degree of convergence (more than 70% of participants selecting 1 or 2 on the Likert scale in either dimension), and few votes in the contextual prioritization instrument.
3. Debatable policy actions: Actions with divergent scores in the two dimensions (i.e., considered applicable but not contextually relevant, or vice versa), different average scores in the rounds, greater dispersion of responses between experts, or borderline mean values (neither high or low priority); or where there were conflicting opinions among the experts regarding prioritization in the urban LAC context.

#### 2.4.3. Validity and Quality of the Information Generated by the Panel

To estimate validity, we evaluated (a) the stability of the panel and the joint group response between dimensions and between rounds by analyzing the change in the coefficient of variation, (b) the time elapsed between rounds, and (c) the complementarity and triangulation of the information.

To assess the quality of information generated, we calculated the degree of experts’ agreement on (a) the clarity and precision of the questionnaire, (b) the effectiveness of the method in obtaining and improving the panel’s opinion, (c) the feedback from the first questionnaire and its usefulness for improving confidence in the responses to the second questionnaire, and (d) satisfaction with participation in the study.

## 3. Results

To generate the required information, we carried out two rounds of evaluation. The expert panel included 21 people in the first round (13 women and 8 men) and 18 people in the second round (12 women and 6 men), meaning that the response rate in the second round was 85.7%. The average age of the participants was 49 years. The participants came from 13 countries in the LAC region (Argentina, Bolivia, Brazil, Chile, Colombia, Costa Rica, Dominican Republic, Ecuador, El Salvador, Mexico, Peru, Uruguay, and Venezuela); Ecuador was slightly overrepresented. Most experts had more than 10 years of experience in the fields of public policy, research, social or community action, and healthcare.

### 3.1. Categorization of Actions

Table 1 summarizes the estimation of consensus and categorization of the actions through triangulation of the information. In the ranking (column A), we observed some degree of stability and coherence for most items over the two rounds. However, some items did change rank between the rounds, or showed divergence between the dimensions. Regarding the degree of convergence of opinion in both rounds (column B), we found differences between the dimensions, with greater consensus on contextual relevance from the first round, while agreement on applicability was much lower in the first round and increased in the second round. When we analyzed the degree of proximity (column C), we found greater dispersion in the dimension of contextual relevance and greater proximity in applicability.

### 3.2. Integration of Information on Contextual Factors That Could Affect the Relevance and Applicability of Policy Actions

The experts considered most recommendations to be highly relevant. In Supplementary Files, Tables S1 and S2 show the comments related to contextual factors that may affect the relevance (S1) and applicability (S2) of the recommended policy actions for promoting healthy eating in urban areas of LAC.

#### 3.2.1. High-Priority Policy Actions

Table 2 lists the high-priority actions. The measures considered high priority by the experts are mostly related to promoting healthy options in schools (8, 13, 14, 15, 17) and publicly funded settings (6, 7). There is also one high-priority recommendation on the sharing of locally produced food (2). The measures are considered highly relevant, especially in agricultural countries, as they combine the guarantee of food sustainability through local production with the provision of healthy products in schools. According to the experts, these health promotion measures should be accompanied by comprehensive programs that promote food education and healthy habits, which can improve the understanding of sustainable practices and encourage lasting changes. The measures were also considered highly applicable, since they can be implemented in contexts that are already publicly regulated and where local actors have more control. Evidence from other LAC countries supports our panel’s positive opinions on these measures; however, specific regulations and political are crucial to address the main barrier to applicability: the commercial interests of ultraprocessed food companies, which enjoy government favoritism owing to their economic impact.

Regarding the promotion of food sharing to improve access to healthy food, there were some differences of opinion. Some experts said that this practice is rooted in the culture and could benefit local production, while others questioned its viability in urban LAC settings owing to the lack of agricultural land. Although they considered food sharing to be crucial for food security, they criticized the lack of government support and suggested that the success of this action would depend on cultural and educational changes. The experts also mentioned the need for large-scale planning to address the limits of medium- and long-term sustainability. Therefore, applicability depends on political will, budget, and efficiency in creating alliances with local producers, markets, and sellers of food products.

In general, the perceptions collected on the high-priority measures cover various aspects, from the need for political will and regulation to practical and economic challenges, with an emphasis on the importance of comprehensive and adaptive strategies.

#### 3.2.2. Low-Priority Policy Actions

Table 3 lists the low-priority policy actions. The measures categorized as low priority are related to improving consumer access to retail stores (1); limiting the number of unhealthy food establishments near schools (4), in the community (5), and in workplaces (21); and regulating the minimum distance of unhealthy food establishments from schools (3). Other actions in this category focus on product labeling (11), positioning of healthy products (18), and providing detailed nutritional information on menus (20).

First, the relevance of these actions in LAC is influenced by the highly informal context of food services and the culture of unhealthy eating. Furthermore, the experts indicated that these types of measures should be introduced at the national level to unify criteria and apply standard regulations both in businesses and in work environments. Here, again, there is a need for considerable political will to develop regulations, monitor compliance, and establish sanctions. Key barriers to implementing these actions are the strong market logic of the food system and resistance by the industry, which holds significant power over product marketing.

Regarding marketing, product labeling has proven effective and applicable at the national level in other countries of the LAC region. The low-priority classification here reflects the fact that some of the panel indicated that labeling should be legislated nationally and applied uniformly throughout the country, so it was not relevant for implementation at local level. Furthermore, it should be conducted in collaboration with the food industry, to unify labeling for all products. In addition, the experts highlighted that healthy foods should not have to compete with unhealthy options in the same outlets. The proposed measure of declaring the calorie content and nutritional value of meals offered in food establishments had low relevance and applicability scores, due to the perceived resistance of small businesses and large chains to its implementation.

The low-priority actions are related to the accessibility of food retailers and regulation of establishments near schools, in the community, and in the workplace. In view of the informal and unhealthy food culture in LAC, these measures require national legislation and face industry resistance in the form of marketing incentives.

#### 3.2.3. Debatable Policy Actions

Table 4 lists the debatable actions. The debatable measures include economic interventions such as taxing sugary drinks and unhealthy foods (12), and the use of incentives and price reductions to promote the purchase of healthy products (16, 19). Other measures were including healthy drinks in nutrition programs (9) and improving the accessibility of nutrition programs, in terms of time and place (10).

There was less consensus among the experts regarding the relevance of these measures on eating habits, with some experts expressing that socioeconomic factors have a stronger influence than knowledge and skills. Therefore, measures like restricting the availability and promotion of unhealthy foods were prioritized. Tax reductions and subsidies could be effective tools if implemented strategically. This would require a comprehensive approach integrating awareness-raising campaigns, education, and collaboration with retailers and food services to ensure accessibility. The experts also mentioned previous strategies that did not succeed owing to lack of cooperation from businesses, such as regulating prices for basic food items.

### 3.3. Assessment of Information Validity and Quality

We observed optimal stability in the panel with fewer than 15% of participants lost between rounds. We had to extend the time period between the two rounds because some experts had limited availability. Regarding stability in the group response, we observed significant complementarity in the quantitative and qualitative information generated by the panel in the open-text boxes to explain their rating. We observed very low or low percentages of variability when categorizing the coefficient of variation (<25% very low variability; 26–50% low), which reflects that a satisfactory level of stability of the group response was reached in the process.

In general, the experts considered that the information generated through the Delphi technique was of high quality. In addition, 95% of participants agreed or strongly agreed that the technique was effective for improving their opinions between rounds, and 82.4% found the feedback from the first questionnaire very useful for improving confidence in their answers to the second questionnaire. Furthermore, almost 90% of the experts agreed or strongly agreed that the questionnaire was clear and precise, and a similar proportion were satisfied or very satisfied with their participation in the study.

## 4. Discussion

This study analyzed the perceived applicability and contextual relevance of local actions recommended by international public health agencies to promote healthy eating in urban settings of the LAC region. We used the Delphi technique to obtain consensus opinions from a group of experts on the contextual relevance and applicability of policy actions to promote healthy diets in local settings.

This technique has been widely used in the health field for forecasting, prognosis, and consensus-building [25,27], based on the principle that collective opinions are superior to individual opinions. Our study met the essential criteria of the technique, including (a) an ordered and monitored iterative process, which allowed the experts to contribute opinions in two rounds, stabilizing their points of view and favoring reflection and adjustment of opinions; (b) participant anonymity, guaranteed through the use of online questionnaires, to prevent participants influencing each other and allow them to change or reaffirm their opinions; (c) controlled feedback, provided from a neutral position; (d) clarification of concepts between rounds; and (e) synthesis of scores and statistical processing of the group response and the individual positions.

Several characteristics of Delphi studies ensure the coherence, validity, and quality of the information generated. This study considered key elements such as the composition of the expert panel, participants’ knowledge and experience, the stability of the panel, the lapse of time between rounds, and the complementarity and triangulation of information. The panel included experts from different countries and sectors to guarantee optimal representativeness, and we obtained a high response rate between rounds, as per the acceptable parameters described in the literature [25,27,28]. The time interval between rounds was considered adequate, with no legislative changes that could affect the results. The experts showed interest and commitment and provided solid arguments. During the process, we modified the wording of some statements to improve understanding of the recommendations. The experts appreciated these changes, which highlights the quality of the information generated, as well as the completeness, robustness, and success of the process. In addition, the triangulation, convergence, and proximity of various indicators enabled a satisfactory level of consensus, which in turn enabled the categorization and prioritization of potential policy actions to promote healthy eating in the LAC region.

The high-priority recommendations include measures related to improving access to and availability of healthier food options. The experts emphasized the feasibility of implementing actions in schools to promote healthy habits. Their opinions are in line with the literature, which highlights the importance of the school environment in promoting healthy behaviors, including healthy eating and physical activity [18,29,30,31,32,33]. However, the experts gave examples of contextual factors that hinder the applicability of these measures, including their uncertain sustainability due to limited budget allocation, changes in government, short-term regulations, reliance on political will, and the lack of rules to regulate the advertising and sale of ultraprocessed foods in school canteens. In addition, the experts highlighted problems with the current programs, including low coverage, lack of monitoring, lack of comprehensiveness, the low nutritional quality of school menus, and the inadequacy of nutrition transitions in some territories. These factors could be mitigated through community participation, food and nutritional education, and the creation of alliances with local producers. In this sense, other experiences show that improvements in infrastructure and equipment, interinstitutional and intersectoral coordination, adoption of adequate and culturally appropriate menus, monitoring, and evaluation, among other factors, could improve these problems and drive positive changes in local food systems [31,33].

The experts indicated that possible legal barriers and pressure from the processed food industry could hinder the implementation of policies that aim to restrict the advertising, promotion, and sale of unhealthy foods in public institutions and specific environments (extending to various public and virtual spaces). The scientific literature provides ample evidence on the obstructive role of the processed food industry, which includes modifying food preferences and purchasing and consumption patterns in favor of foods with low nutritional quality [17,32,34,35,36]. Therefore, the successful application of this policy depends on effective control, surveillance, and sanctions. Expanding the availability of healthy foods in schools, preschools, and other settings is crucial, but it is necessary to overcome challenges such as the lack of uniform ethical codes for advertisers and the food industry in different countries [17,21,32,36,37]. Ultraprocessed foods are also commonly available in vending machines, particularly in schools. The expert panel discussed vending machines in relation to environmental sustainability and healthy options; they pointed out that offering healthy options is impractical due to conservation and biosafety problems. Some countries have prohibited vending machines in schools (France and the UK) or have recommended limiting minors’ access to these machines, restricting advertising, and promoting a balanced diet (Spain) [21,38].

Regarding the promotion of food and nutrition education in school classes, there was some disagreement about how to integrate this plan into the curriculum. In this sense, the literature provides several examples,, either as programs integrated into formal curricula (as independent subjects or with cross-cutting content in several subjects), or as specific projects, or extracurricular activities, highlighting/emphasizing that any program should be contextualized and based on local nutritional needs [29,30,32,33].

The panel considered that promoting food-sharing networks, community gardens and greenhouses, and farmers markets could help not only to reduce overweight and obesity, but also to support local production and community participation. Positive opinions on this policy focused on agroecology, gender equality, circular economy, fair trade, and food and nutritional security. These initiatives, which are being integrated into development programs and plans in the LAC region, require local leadership and the support of empowered community networks to succeed [8,39]. However, the expert panel mentioned several obstacles, including lack of planning and infrastructure, time and space limitations, government resistance, lack of political will to adapt land use regulations, and medium- to long-term sustainability challenges.

Considering the high rates of poverty and marginality in certain sectors of Latin American cities [16], many food-related actions and programs in the LAC region currently focus on improving economic and physical accessibility of food through subsidies or welfare benefits to satisfy the needs of the most vulnerable population [40], although some countries have begun to adopt broader approaches [41]. Positive experiences with the use of coupons for fresh foods in Chile, Argentina, Mexico, and Uruguay emphasize the importance of focusing spending on healthy products. Subsidies are considered crucial to guarantee access to healthier foods [19]. Actions to guarantee the right to a healthy diet must extend beyond temporary solutions. According to our experts, the approach must guarantee food sovereignty to protect peoples’ right to a healthy, nutritious, culturally appropriate, and sustainable diet [8].

On the other hand, the experts considered taxation of sugary drinks and foods high in fat and sugar to be an irrelevant and ineffective action for promoting healthy diets; they questioned the evidence on the measure and its applicability at the local level. They argued that fiscal measures require a central or national response, which could generate contradictions if implemented locally. Some countries in the LAC region have faced significant obstacles when trying to implement these measures [17,37,42], although there are cases of successful changes in legislation that demonstrate the direct influence of citizens’ concerns on government decisions [43,44].

Although front-of-pack labeling has been implemented and promoted in the LAC region [8,34,37,45], the experts considered this a low-priority measure, owing partly to experiences of implementation difficulties, lack of impact on consumption, and industry obstacles in some countries. The recommendation of including nutritional information on menus in restaurants and other outlets was also considered a low priority, although it would increase transparency [21].

Another low-priority policy action was zoning of food establishments, because they are difficult to control and often use informal labor, and because many food establishments in LAC cities are illegal. In other countries, this measure has successfully controlled the number of fast-food establishments near schools and the minimum distance of fast-food establishments from schools [21]. In this sense, according to our study participants, food retailers and outlets play a key role in promoting healthier eating practices at the local level, and they must be involved in the implementation of public policies to improve diets.

In general, we found that the successful implementation of nutrition policies requires official adoption by relevant government agencies, cross-sector governance, and the participation of local partners and communities. The panel emphasized the importance of framing local actions within national legislation to guarantee the viability, continuity, and financing of the actions, and to implement comprehensive, planned, budgeted, coordinated, monitored, and regularly evaluated strategies.

This study has several limitations inherent to its methodology. As a Delphi study, it is subject to participant assessment biases. In this sense, there could be a potential sampling bias, given that a small proportion of participants were selected based on personal contacts of the research team. Additionally, as research focusing on a specific context, the results could influenced by significant changes in the conditions of LAC countries. For example, a shift from a biomedical model to a more comprehensive healthcare model could mean that certain policies become more prioritized than others. On the other hand, there are other factors not considered in this study that could impact the feasibility or implementability of these policies, such as the geographical limitation and background of the experts, as well as individual factors, cultural elements, and support networks.

However, this study followed a rigorous process to assess the opinions of experts from several LAC countries and could serve as a valuable “roadmap” for policy actions aimed at improving dietary habits among Latin American populations. With the rapid growth and transformation of the food system in LAC countries (the rise of supermarkets, large processors, fast-food chains, and food logistics companies [17]), it is necessary to implement actions that alleviate or delay negative effects in urban areas. We hope that this document can help to guide local decisionmakers who wish to implement measures to promote healthier eating in the LAC region.

## 5. Conclusions

We identified various contextual factors that may affect the relevance and applicability of policy actions for the promotion of healthy eating in urban areas of the LAC region. It is necessary to contextualize these actions and analyze the related factors to guarantee their successful implementation and improve local food environments and, thus, people’s health.

This study encourages local health actors to focus on the eight policy actions proposed by public health agencies, which local experts have identified as high priority by experts due to their feasibility and better alignment with the current contexts of LAC. At the same time, it underscores the importance of further investigating, through subsequent research, the contextual factors that undermine the implementation of other public policies aimed at promoting healthy eating.

## Figures and Tables

**Table 1:** Estimation of consensus and categorization of policy actions in the two rounds.

Ítem	Ranking ^a^				Degree of Convergence of Opinions (%) ^b^				Degree of Proximity ^c^		Contextual Prioritization ^d^	Categorization of Recommendations ^e^
	1st Round		2nd Round		1st Round		2nd Round					
	CR	A	CR	A	CR	A	CR	A	CR	A		
1	17	17	16	14	85.7	42.9	83.3	50.0	0.0	−0.1	2	**Low Priority**
2	14	14	7	5	90.5	61.9	88.9	72.2	0.3	0.3	10	**High Priority**
3	19	20	20	21	66.7	33.3	66.7	83.3	0.0	0.4	0	**Low Priority**
4	21	21	21	20	57.1	23.8	66.7	72.2	−0.1	0.1	1	**Low Priority**
5	20	16	18	19	81.0	57.1	66.7	72.2	0.0	0.5	0	**Low Priority**
6	8	9	2	9	95.2	66.7	94.4	66.7	0.0	0.3	7	**High Priority**
7	9	3	5	3	90.5	71.4	94.4	77.8	0.1	0.3	7	**High Priority**
8	2	5	3	11	100	81.0	94.4	77.8	−0.1	−0.3	9	**High Priority**
9	12	4	12	1	76.2	71.4	77.8	76.2	−0.2	0.3	2	**Debatable**
10	18	7	11	4	76.2	71.4	83.3	72.2	0.2	0.3	4	**Debatable**
11	15	15	15	15	85.7	61.9	77.8	50.0	0.0	0.4	4	**Low Priority**
12	11	8	6	17	85.7	57.1	94.4	50.0	0.1	0.0	2	**Debatable**
13	1	1	1	2	100	81.0	100	72.2	0.1	−0.2	7	**High Priority**
14	3	2	4	10	100	76.2	94.4	76.2	0.4	0.1	9	**High Priority**
15	6	10	10	8	95.2	66.7	83.3	72.2	−0.6	0.1	4	**High Priority**
16	13	12	17	7	90.5	66.7	72.2	66.7	−0.4	0.3	1	**Debatable**
17	4	6	8	6	100	76.2	94.4	72.2	−0.4	−0.2	11	**High Priority**
18	10	11	13	13	90.5	61.9	83.3	61.1	−0.2	−0.1	2	**Low Priority**
19	7	18	9	18	95.2	42.9	83.3	38.9	−0.2	−0.1	3	**Debatable**
20	16	19	19	16	81.0	47.6	72.2	44.4	0.0	0.5	2	**Low Priority**
21	5	13	14	12	100	61.9	94.4	50.0	−0.3	0.2	3	**Low Priority**

Note: CR: contextual relevance; A: applicability. ^a^ Position 1 in the ranking shows the highest rated items in green (high degree of applicability or high level of contextual relevance); ^b^ Gray shows the degree of agreement with criteria >70% of grouping of the 4/5 or 1/2 ratings; ^c^ Evaluation of the difference in standard deviation between the first and second round; ^d^ Number of times voted as the main recommendation in context LAC. ^e^ Recommendations categorized as High Priority are shown in green, Low Priority in red, and debatable in yellow.

**Table 2:** Policy actions designated by the panel as High Priority.

Policy Action
Item 2: Promote food sharing networks, community gardens/greenhouses, and farmer’s markets to help address food insecurity and improve access to locally grown healthy food.
Item 6: Authorities should ensure publicly funded venues (e.g., museums, recreational centers), especially those frequented by children and young people, resist sponsorship or product placement from companies associated with foods and beverages high in fat, sugar, or salt.
Item 7: Competent authorities should ensure that places using public money to procure food and beverages provide a range of healthier and more affordable options (even in vending machines), e.g., school visits to museums, sports centers, cinemas, and theme parks.
Item 8: Use welfare benefits and wider schemes to supplement the family food budget and improve eating patterns, e.g., free school meals, free school fruit, and vouchers for healthy food outlets.
Item 13: Adopt school meal policies that ensure that school breakfasts or lunches meet specific nutrition requirements and offer taste tests of new menu items.
Item 14: Introduce school programs that provide fruit and vegetables to pupils during breaktimes.
Item 15: Set up attractive displays of fruits and vegetables in school canteens.
Item 17: Use class time to encourage healthy eating and physical activity. It can be taught as a specific subject (e.g., physical education), or as part of other subjects (e.g., science, home economics, mathematics, agriculture), or, ideally, as a combination of both.

**Table 3:** Policy actions designated by the panel as Low Priority.

Policy Actions
Item 1: Ensure local accessibility, either on foot or by public transport, to retailers (supermarkets, corner shops, street markets, and small independent shops) that sell healthy food and drink.
Item 3: Regulate the distance from schools and the opening hours of takeaway and other food outlets that specialize in foods high in fat, salt, or sugar.
Item 4: Set limits for the number of takeaway and junk food outlets in a given area, particularly those near schools.
Item 5: Authorities should systematically consider healthier eating options when reviewing applications for new food outlets.
Item 11: If within existing competencies, local authorities should introduce a simple front labeling system for packaged foods, with one single and easy-to-understand label, established independently of the food industry and which guarantees that the food in question is among the healthiest options in its group. Where possible, include in the regulation sanctions for companies that do not comply with the standards.
Item 18: Use of posters, verbal prompts, and product positioning to promote healthier food and drink choices among the population at food outlets (retailers, restaurants, vending machines, and other food sources in the community).
Item 20: At food outlets that prepare recipes, include details in menus on the calorie content of meals to help consumers make an informed choice. If the nutritional value of recipes is not known, they should list ingredients and describe the cooking methods used.
Item 21: At workspaces, promote healthier food and drink choices in staff and client restaurants, hospitality suites, vending machines, and shops by using posters, verbal prompts, lower pricing, and the positioning of products.

**Table 4:** Policy actions designated by the panel as debatable.

Policy Actions
Item 9: Incorporate a healthy beverage recommendation into nutritional standards that serve as a guide for government nutrition programs or the food industry.
Item 10: When public nutritional education programs are offered, ensure they are scheduled at times that suit people with children (or provide a crèche), fit with diverse working hours, and take place in socially acceptable venues (such as community centers) that are accessible locally, either on foot or via public transport.
Item 12: If within existing competencies, local authorities should introduce taxes on sugary drinks and products high in fat and sugar in order to reduce their consumption.
Item 16: Use posters, verbal prompts, lower (tactical) pricing, and product positioning to promote healthier food and drink choices in canteens/bars and vending machines.
Item 19: Food outlets (retailers, restaurants, vending machines, and other food sources in the community) should price healthier foods and beverages at lower costs and use incentives (such as promotional offers) to promote healthier choices.

## Data Availability

The data described in the study are not publicly available due to privacy and ethical restrictions but may be made available upon request. A detailed report with more extensive results and the complete list of recommendations in Spanish is available in the Zenodo repository: “Final Report: Delphi Study on the Contextualization of Recommendations to Promote Healthy Diets in Urban Latin American Settings” (Spanish version) DOI: 10.5281/zenodo.11032155.

## Supplementary Materials

The following supporting information can be downloaded at: https://www.mdpi.com/article/10.3390/nu16234017/s1, Table S1: Synthesis of qualitative information regarding the perceived contextual relevance of political actions to promote healthy diets in urban settings in the LAC region; Table S2: Synthesis of qualitative information regarding the perceived applicability of political actions to promote healthy diets in urban settings in the LAC region.

## Appendix A

List of the members of the Working Group of Public Policies to Promote Healthy Diets in Urban Contexts in Latin America and the Caribbean:

David Acurio Páez, Universidad de Cuenca, Ecuador; Fátima Aguayo Arteaga, Medicina Familiar, Bolivia; Vhania Batista Rodríguez, Sistema Dominicano de Salud. República Dominicana; Anselmo Cancino, Departamento de Promoción de la Salud y Participación Ciudadana. Facultad Latinoamericana de Ciencias Sociales, FLACSO, Chile; Virginia Cardozo Rufo, Intendencia de Montevideo, Uruguay; Karla Patricia Chiquillo Sosa, Ministerio de Salud de El Salvador; Jaime Delgado Zegarra, Universidad de San Martín de Porres, Perú; Mayra Patricia Erazo Navas, Ministerio de Salud de El Salvador; Wilma Freire, Sociedad Científica, diversos países LAC; Sandra Gamboa, Escuela de Salud Pública de la Universidad de Costa Rica; Verónica Beatriz González, Estaciones Saludables. Gobierno de la Ciudad de Buenos Aires, Argentina; Maritza Landaeta-Jiménez, Fundación Bengoa, Alimentación y Nutrición, Venezuela; Eduardo Nilson, Fundación Oswaldo Cruz (Fiocruz), Brasil; José Andrés Ocaña Navas, Instituto de Salud Pública, Pontificia Universidad Católica del Ecuador; Gabriela Rivas, gestión de política pública de nutrición, varios países LAC; Valerin Saurith, Organización indígena; Universidad Pedagógica y Tecnológica de Colombia; María Soledad Tapia, Universidad Central de Venezuela; Fundación “5aldía Venezuela”; Academia de Ciencias Físicas, Matemáticas y Naturales. Venezuela; Gabriela Vargas Romero, Cultiva Ciudad y Huerto Tlatelolco, México; José Julio Villalba Vásquez, Instituto de Salud Pública, Pontificia Universidad Católica del Ecuador, Ecuador; Matías Villatoro Reyes, Ministerio de Salud de El Salvador.

## Appendix B

**Table A1:** Detailed information about the recruitment process of the panel of experts ^a^.

Predetermined Criteria	Requirements
**1. Number of Participants**	A minimum of 15 participants per round.
**2. Geographical Scope**	(a) Representation from at least 8 LAC countries. (b) No more than 3 participants from the same country (c) Participation from at least two countries in each area of expertise. (d) Residency in an LAC country.
**3. Gender Equity**	Achieved a gender balance of 40/60.
**4. Competence**	Experts were required to have competence in aspects related to public health, public policy, health systems, dietary habits promotion, health promotion, disease prevention, food sovereignty, and healthy eating.
**5. Experience**	(a) Political: Individuals working in political decision-making roles or institutions, or involved in implementing public policies at local, national, or international levels. (b) Academic: University researchers, members of research institutes, scientific societies, or academic groups. (c) Community: Recognized community members engaged in voluntary actions related to health promotion, disease prevention, food sovereignty, and healthy eating, including work with NGOs. (d) Healthcare: Professionals in the health sector or health systems, such as family doctors, intensivists, nurses, nutritionists, and health managers.
**6. Availability**	Participants were selected based on their ease of contact, high motivation, willingness, and commitment to participate in all phases of the study.
**7. Impartiality**	Individuals with conflicts of interest were excluded. Only those with minimal or no conflict regarding the study’s outcomes were included.

^a^ An expert was defined as an individual whose situation and resources enable them to contribute positively to the contextualization and prioritization of policy actions aimed at promoting healthy diets in urban settings within LAC countries. Experts included specialists, affected individuals, collaborators, and facilitators.

## References

[1] World Health Organization (2013). Global Action Plan for the Prevention and Control of Noncommunicable Diseases 2013–2020.

[2] World Obesity Federation (2020). Obesity: Missing the 2025 Global Targets. Trends, Costs and Country Reports.

[3] Organización Panamericana de la Salud (2019). Las ENT de Un Vistazo. Mortalidad Por Enfermedades No Transmisibles y Prevalencia de Sus Factores de Riesgo En La Región de Las Américas.

[4] Congdon P. (2019). Obesity and Urban Environments. Int. J. Environ. Res. Public Health.

[5] Organización Panamericana de la Salud (2019). Enfermedades No Transmisibles En La Región de Las Américas: Hechos y Cifras.

[6] Organización Panamericana de la Salud (2019). Indicadores Básicos 2019. Tendencias de La Salud En Las Américas.

[7] Fondo de las Naciones Unidas para la Infancia (2023). Crece La Ola de Sobrepeso En La Niñez. ¿Demasiado Tarde Para Revertir La Marea En América Latina y El Caribe?.

[8] FAO, FIDA, OPS, WFP, UNICEF (2020). Panorama de La Seguridad Alimentaria y Nutricional En América Latina y El Caribe. Seguridad Alimentaria y Nutricional para Los Territorios más Rezagados, 2020.

[9] FAO, OPS, WFP, UNICEF (2019). Panorama de La Seguridad Alimentaria y Nutricional En América Latina y El Caribe.

[10] United Nations System Standing Committee on Nutrition (2018). Enfermedades No Transmisibles, Dietas y Nutrición.

[11] Rivera J., Barquera S., Campirano F., Campos I., Safdie M. (2002). Epidemiological and Nutritional Transition in Mexico: Rapid Increase of Non-Communicable Chronic Diseases and Obesity. Public Health Nutr..

[12] Jiménez Acosta S., Rodríguez Suárez A., Díaz Sánchez M. (2013). La Obesidad En Cuba. Una Mirada a Su Evolución En Diferentes Grupos Poblacionales. Rev. Cuba. Aliment. Nutr..

[13] Sarmiento O.L., Parra D.C., González S.A., González-Casanova I., Forero A.Y., Garcia J. (2014). The Dual Burden of Malnutrition in Colombia. Am. J. Clin. Nutr..

[14] Martínez R., Mejía C., Espíndola E. (2024). The Cost of the Double Burden of Malnutrition. Main Social and Economic Impacts in Eight Latin American Countries.

[15] WHO (2017). Global Nutrition Targets 2025: Policy Brief Series.

[16] Comisión Económica para América Latina y el Caribe (CEPAL) (2019). Panorama Social de América Latina y El Caribe.

[17] Popkin B.M., Reardon T. (2018). Obesity and the Food System Transformation in Latin America. Obes. Rev..

[18] FAO, OMS, UNICEF, FIDA, WFP (2023). El Estado de La Seguridad Alimentaria y La Nutrición En El Mundo 2023. Urbanización, Transformación de los Sistemas Agroalimentarios y Dietas Saludables a lo Largo del Continuo Rural-Urbano.

[19] WHO (2014). Comprehensive Implementation Plan on Maternal, Infant and Young Child Nutrition.

[20] Naciones Unidas Transformar Nuestro Mundo: La Agenda 2030 Para El Desarrollo Sostenible. Proceedings of the Asamblea General, 4ª Sesión Plenaria, A/RES/70/1.

[21] Lee A.J., Cullerton K., Herron L.M. (2021). Achieving Food System Transformation: Insights From A Retrospective Review of Nutrition Policy (In)Action in High-Income Countries. Int. J. Health Policy Manag..

[22] International Network for Food and Obesity/Non-Communicable Diseases Research Monitoring and Action Support INFORMAS. https://www.informas.org/.

[23] Blasco-Blasco M., Puig-García M., Piay N., Lumbreras B., Hernández-Aguado I., Parker L.A. (2020). Barriers and Facilitators to Successful Management of Type 2 Diabetes Mellitus in Latin America and the Caribbean: A Systematic Review. PLoS ONE.

[24] Chilet-Rosell E., García M.P., Lumbreras B., Valero M.P., Aguado I.H., Parker L.A. (2024). Promoting Physical Activity and Healthy Diets by Modifying the Social and/or Physical Environment at Local Level: A Scoping Review of Evidence-Based Policy Actions. [Version 1; Peer Review: Awaiting Peer Review]. Open Res. Eur..

[25] Landeta J. (1999). El Método Delphi. Una Técnica de Previsión Para La Incertidumbre.

[26] Brady S.R. (2015). Utilizing and Adapting the Delphi Method for Use in Qualitative Research. Int. J. Qual. Methods.

[27] Diamond I.R., Grant R.C., Feldman B.M., Pencharz P.B., Ling S.C., Moore A.M., Wales P.W. (2014). Defining Consensus: A Systematic Review Recommends Methodologic Criteria for Reporting of Delphi Studies. J. Clin. Epidemiol..

[28] Fernández-Ávila D.G., Rojas M.X., Rosselli D. (2020). Delphi Method in Rheumatology Research: Are We Doing Well?. Rev. Colomb. Reumatol..

[29] Fundación Española de la Nutrición (2013). Libro Blanco de La Nutricion En España.

[30] O’brien K.M., Barnes C., Yoong S., Campbell E., Wyse R., Delaney T., Brown A., Stacey F., Davies L., Lorien S. (2021). School-Based Nutrition Interventions in Children Aged 6 to 18 Years: An Umbrella Review of Systematic Reviews. Nutrients.

[31] FAO, Agencia Brasileña de Cooperación del Ministerio de Relaciones Exteriores (ABC/MRE), Fondo Nacional de Desarrollo de la Educación del Ministerio de Educación (FNDE/MEC) (2023). Escuelas Sostenibles. Orientaciones Conceptuales y Metodológicas.

[32] World Health Organization (2020). Nutrition Action in Schools: A Review of the Evidence Related to the Nutrition-Friendly Schools Initiative.

[33] FAO (2022). Educación Alimentaria y Nutricional En Las Escuelas. Un Libro Blanco Sobre el Estado Actual, Principios, Desafíos y Recomendaciones para Países de Ingresos Bajos y Medianos.

[34] Mialon M., Charry D.A.G., Cediel G., Crosbie E., Scagliusi F.B., Tamayo E.M.P. (2021). “I Had Never Seen so Many Lobbyists”: Food Industry Political Practices during the Development of a New Nutrition Front-of-Pack Labelling System in Colombia. Public Health Nutr..

[35] Mialon M., Da Silva Gomes F. (2019). Public Health and the Ultra-Processed Food and Drink Products Industry: Corporate Political Activity of Major Transnationals in Latin America and the Caribbean. Public Health Nutr..

[36] Baker P., Machado P., Santos T., Sievert K., Backholer K., Hadjikakou M., Russell C., Huse O., Bell C., Scrinis G. (2020). Ultra-Processed Foods and the Nutrition Transition: Global, Regional and National Trends, Food Systems Transformations and Political Economy Drivers. Obes. Rev..

[37] Letona P. (2013). Estudio Exploratorio Sobre La Promoción y Publicidad de Alimentos y Bebidas No Saludables Dirigida a Niños En América Latina y El Caribe.

[38] Agencia Española de Seguridad Alimentaria (2005). Estrategia Nacional de Nutrición, Actividad Física y Prevención de La Obesidad (NAOS). Invertir la Tendencia de la Obesidad.

[39] Monroy K. (2016). Agricultura Urbana Como Alternativa de Seguridad Alimentaria y Nutricional. Tesis de Grado, Pontificia Universidad Javeriana.

[40] Gilardon E.O.A. (2016). Una Evaluación Crítica de Los Programas Alimentarios En Argentina. Salud Colect..

[41] Frente Parlamentario contra el Hambre (2003). El Plan de Seguridad Alimentaria, Nutrición y Erradicación Del Hambre de La CELAC y Los Frentes Parlamentarios Contra El Hambre. La Experiencia Argentina.

[42] Pan American Health Organization (2020). Sugar-Sweetened Beverage Taxation in the Region of the Americas.

[43] Webpage Red Papaz Plataforma de Movilización Ciudadana. https://entretodos.redpapaz.org/es/movilizaciones.

[44] (2022). Ley 2277 de 2022. Por Medio de La Cual Se Adopta Una Reforma Tributaria Para La Igualdad y La Justicia Social y Se Dictan Disposiciones. Título X “Impuestos Saludables” del 13 dic de 2022; Colombia. https://www.funcionpublica.gov.co/eva/gestornormativo/norma_pdf.php?i=199883.

[45] Alianza por la Nutrición Infantil, Cámara de la Industria de Alimentos de la ANDI, Asociación ABACO, Asociación de Bancos de Alimentos de Colombia, Fundación Éxito (2018). Revisión Documental de Intervenciones Nutricionales Exitosas a Nivel Internacional y Nacional.

