# Supplementary material for: Delphi Study on the Contextualization of Recommendations for Promoting Healthy Eating in Urban Settings of Latin America and the Caribbean
Source: Nutrients. 2024 Nov 24;16(23):4017. doi: 10.3390/nu16234017 (PMC11643776; doi:10.3390/nu16234017)
Supplement: Supplementary file 1 [file nutrients-16-04017-s001.zip › nutrients-3308857-supplementary.pdf]

Supplementary Files:

Table S1. Synthesis of qualitative information regarding the perceived contextual relevance of political actions to promote healthy diets in Urban Settings in the LAC region.

Table S2. Synthesis of qualitative information regarding the perceived applicability of political actions to promote healthy diets in Urban Settings in the LAC region.

Table S1. Synthesis of qualitative information regarding the perceived contextual relevance of political actions to promote healthy diets in Urban Settings in the LAC region.

Rank	Nº	Recommendation	Perception of contextual relevance.
1	13	Adopt school meal policies that ensure school breakfasts or lunches meet specific nutrition requirements and offer taste tests of new menu items.	A feasible, relevant, and essential measure (for both public and private schools). The products should be locally sourced and take into consideration the nutritional and epidemiological transition circumstances of students in each region.<br>It is not relevant if school days are not full-time.
2	6	Authorities should ensure publicly funded venues (e.g., museums, and recreational centers), especially those frequented by children and young people, resist sponsorship or product placement from companies associated with foods and beverages high in fat, sugar, or salt.	- A relevant measure that should be applied to all public spaces funded with public funds, regardless of their audience (with a priority on children and adolescents).<br>- Most experts comment that it is a highly relevant measure as it requires precise regulations, is easy to implement, and could be efficient. However, commercial interests could complicate the measure.<br>- Public policy can be planned at the local level, by autonomous decentralized governments, and at the national level, by the health authority or the Ministry of Health.
3	8	Use welfare benefits and wider schemes to supplement the family food budget and improve eating patterns. e.g., free school meals, free school fruit and vouchers for healthy food outlets.	- It is considered a viable, useful, and highly relevant measure, especially for social groups with limited access to food.<br>- This measure should be accompanied by food education.
4	14	Introduce school programs that provide fruit and vegetables to pupils during break-time.	- A highly relevant and necessary measure already present in some countries. It should preferably come from local and ecological production, especially in agricultural-based countries.<br>- It is necessary to monitor the safety of food.<br>- This recommendation could strengthen the role of food in promoting healthy diets and healthy eating habits.
5	7	Competent authorities should ensure that places using public money to procure food and beverages provide a range of healthier and more affordable options (even in vending machines). e.g., school visits to museums, sports centers, cinemas, and theme parks.	- This measure has received high ratings and is seen as a good proposal.<br>- Some experts who rated the relevance lower suggest the need for educating the population about making healthier food choices.<br>- This measure is considered a challenge, especially when compared to the financing of ultra-processed products.
6	12	If within existing competencies, local authorities should introduce taxes on sugary drinks and products high in fat and sugar to reduce their consumption.	- It is a highly relevant measure, although there are differences of opinion among experts regarding the evidence for this measure.

Rank	Nº	Recommendation	Perception of contextual relevance.
7	2	Promote food sharing networks, community gardens/greenhouses, and farmers' markets to help address food insecurity and improve access to locally grown healthy food.	- There was not much consensus on relevance. Some consider it a very common and deeply rooted practice in their culture, promoting local production and community participation, strengthening the local economy, and aligning positively with agroecology, gender equality, circular economy, and fair trade.<br>- On the other hand, there were concerns about the existence of a food-sharing culture, the absence of market spaces and infrastructure offering products from local producers in urban Latin America, and doubts about the feasibility of making these exchange mechanisms a public policy option.<br>- It is recognized as a relevant measure for achieving Food and Nutritional Security, but there is little momentum from municipal governments.<br>- It is applicable and contextually relevant as long as it is complemented with community-based food education, social and cultural transformations that empower community networks with these initiatives.<br>- It is an important measure in times of syndemic challenges involving overweight, obesity, and climate change.
8	17	Use class time to encourage healthy eating and physical activity. It can be taught as a specific subject (e.g., physical education) or, as part of other subjects (e.g., science, home economics, mathematics, agriculture), or ideally as a combination of both.	- The relevance of this measure is reflected as it is effective when combined with policies for healthy school food environments and adequate physical activity, especially in low-income countries.<br>- Highly relevant and should be incorporated into education as part of comprehensive education. However, it is mentioned that teachers may not be adequately trained in nutrition topics.<br>- In some countries, the introduction of nutrition education is already mandated by law, and there are methodological guidelines for healthy eating from the Ministries of Education.<br>- It is essential that nutrition education from an early age includes culinary education. However, post-pandemic, schools are prioritizing the recovery of usual curriculum content.
9	19	Food outlets (retailers, restaurants, vending machines and other food sources in the community) should price healthier foods and beverages at lower costs and use incentives (such as promotional offers) to promote healthier choices.	- A measure that has been rated as very relevant but not necessarily applicable in the Latin American and Caribbean context.<br>- It is necessary to assess where to invest these incentive resources, considering the variety of food supply sources in specific environments and the percentage of food purchase from these sources (in many places, traditional markets still surpass retailers).<br>- This measure would stimulate healthy consumption but would require an agreement between consumers and traders.
10	15	Set up attractive displays of fruits and vegetables in school canteens.	-Relevant for supporting local producers. It is a strategy that should be accompanied by public policies and surveillance and monitoring systems.

Rank	Nº	Recommendation	Perception of contextual relevance.
11	10	When public nutritional education programs are offered, ensure they are scheduled at times that suit people with children (or provide a crèche), fit with diverse working hours and take place in socially acceptable venues (such as community centers) that are accessible locally, either on foot or via public transport.	- It has been mentioned that food education should be present in the programs, but environmental modification is considered to be of greater relevance.
12	9	Incorporate a healthy beverage recommendation into nutritional standards that serve as a guide for government nutrition programs or the food industry.	- Considered a highly relevant measure, but there is a debate among experts about the use and impact of dietary guidelines. Some comments highlight that visual guides are a great tool, while others point out their limited real impact on consumption, preferring labeling policies and advertising regulation.<br>- Because some national dietary guidelines are based on foods rather than nutrients, it is emphasized that the measure should consider recommendations regarding the degree of industrial processing of beverages. Using a single nutritional profile, such as calorie limits or critical nutrients (as often proposed in some dietary guidelines), may open the door to unwanted food reformulations, such as the use of non-caloric sweeteners and other food additives.<br>- Relevant, but it should be part of a comprehensive strategy, including consumer education, campaigns, and public policies to guide nutrition programs for greater impact (labeling alone may not suffice).
13	18	Use of posters, verbal prompts, and product positioning to promote healthier food and drink choices among the population at food outlets (retailers, restaurants, vending machines, and other food sources in the community).	- It would be ideal. It is considered a relevant measure, but there is a need for mass education and social participation processes to make it culturally accepted.
14	21	At workspaces, promote healthier food and drink choices in staff and client restaurants, hospitality suites, vending machines, and shops by using posters, verbal prompts, lower pricing and the positioning of products.	- It would be a positive strategy. There are previous experiences of fruit consumption offered by public institutions. This can be achieved through strategic public-private partnerships and coordination with corporate medical services.<br>- It is necessary to promote the culture of food planning and the availability of healthy foods in both the public and private sectors. There are decrees to fulfill these actions.<br>- It is essential to consider that, in some Latin American countries, the labor context is characterized by high labor informality, long working hours, great instability, and food insecurity, making it challenging to implement this strategy.<br>- It is crucial to recognize that in low- and middle-income contexts, the food system is still family-based and small-scale farming, making the application of evidence generated in high-income countries a challenge.
15	11	If within existing competencies, local authorities should introduce a simple front labelling system for packaged foods, with one single and easy to understand label, established independently of the food industry and which guarantees that the food in question is among the healthiest options in its group. Where possible, include in the regulation sanctions for companies that do not comply with the standards.	- Front-of-pack warning labels are considered an effective measure with a significant impact on the recognition of unhealthy foods and their influence on food purchases in Latin America.<br>- Relevant for highlighting healthy foods.<br>- Local public policies require support and reinforcement from national measures based on evidence.

Rank	Nº	Recommendation	Perception of contextual relevance.
16	1	Ensure local accessibility, either on foot or by public transport, to retailers (supermarkets, corner shops, street markets, and small independent shops) that sell healthy food and drink.	- It has been rated as a relevant recommendation at the urban level.<br>- It is recognized that in the urban context of Latin America and the Caribbean, small retailers play a significant role, as several Latin American countries maintain the culture of neighborhood stores, proximity, and a wide network of small markets, small-scale sales, food stalls, convenience stores, and informal food vendors.<br>- It is noted that there is usually greater access to unhealthy products in these stores.<br>- Recognized as an important action to "avoid food deserts and improve the availability of affordable and healthy food options, conveniently located with adequate access routes."
17	16	Use posters, verbal prompts, lower (tactical) pricing and product positioning to promote healthier food and drink choices in canteens/bars and vending machines.	- This measure could support the local industry. Some participants believe that economic reduction is more important than promoting with signs.
18	5	Authorities should systematically consider healthier eating options when reviewing applications for new food outlets.	- It is considered a relevant, very necessary measure, although several experts highlight the limitation of the high informality of food supply in various Latin American countries.<br>- Some participants commented that it could be a good option and could help local authorities take actions to reduce taxes on the sale of healthy foods and control the sale of unhealthy products. However, this measure may have little relevance in contexts where the culture is based on unhealthy eating.<br>- It is emphasized that, within the term "healthy," it should also consider foods from fair trade, with a low carbon footprint, clean foods, and free from agrochemicals.
19	20	At food outlets that prepare recipes include details in menus on the calorie content of meals to help consumers make an informed choice. If the nutritional value of recipes is not known, they should list ingredients and describe the cooking methods used.	- This measure has been rated as a measure with low relevance. The comments indicate that: a) there is little or unknown evidence on the impact of this measure; b) in some countries, it did not work due to industry pressure; c) most people tend to be unaware of calorie values, requiring education; d) this measure could have psychological effects (especially in eating disorders, emphasizing calorie values may give too much importance to nutrients); and e) kitchen staff may be reluctant to publish their recipes.<br>- Some participants emphasize that this measure can be very positive for showing menu details and calorie content. However, it is suggested that the focus should be on promoting healthy foods rather than nutrients, and ideally, encouraging home-cooked meals.
20	3	Regulate the distance from schools and the opening hours of take-away and other food outlets that specialise in foods high in fat, salt or sugar.	- Although it received a low ranking, the comments regarding its relevance are positive.<br>- Most participants agree that fast food or high-sugar food outlets are often located near schools, and there are very few sugar-free products available.<br>- It is considered a relevant measure with a significant impact on health, but it has not been prioritized as a public policy in Latin America and the Caribbean.

Rank	Nº	Recommendation	Perception of contextual relevance.
21	4	Set limits for the number of take-away and junk food outlets in a given area, particularly those near schools.	- This measure is recognized as relevant and potentially positive for the health of schoolchildren. It is emphasized that regulating the market for unhealthy products, especially those targeted at infants and schoolchildren, is essential.<br>- It is currently a relevant measure in urban areas in Latin America and the Caribbean due to the widespread presence of legal, illegal, and informal food outlets, as well as the delivery of junk food by various companies. These practices have a significant impact.<br>- Some participants questioned the relevance of this measure, considering it a challenge, and suggested giving priority to other policies, such as: a) tackling the smuggling of ultra-processed foods inside and outside schools; b) promoting health-oriented schools; c) ensuring healthy school meals; d) eliminating bars and kiosks in schools; and e) promoting physical activity and mental health.

Table S2: Synthesis of qualitative information regarding the perceived applicability of political actions to promote healthy diets in Urban Settings in the LAC region.

Rank	N°	Recommendation	Integration of qualitative information on the perception of applicability
1	9	Incorporate a healthy beverage recommendation into nutritional standards that serve as a guide for government nutrition programs or the food industry.	- This initiative requires significant technical development and complex dissemination, as well as a budget.<br>- Applicable whenever it is regulated by law. Some countries already have legislation in this regard. The regulation should establish the mandatory and transparent disclosure of critical ingredients in beverages.<br>- Some guidelines already recommend drinking water (e.g., the "Jarra del Buen Beber" in Mexico, experiences in Argentina, Chile, and Ecuador). It is suggested that this should be the only recommended beverage.
2	13	Adopt school meal policies that ensure school breakfasts or lunches meet specific nutrition requirements and offer taste tests of new menu items.	- Highly applicable measure with positive experiences in various countries, although it is not implemented in some, lacks continuity or the programs are deficient and need improvement.<br>- There is a need for national regulations and political will to implement them, along with sufficient budget allocation.<br>- This policy should outlive changes in government.<br>- This recommendation requires public management and monitoring.
3	7	Competent authorities should ensure that places using public money to procure food and beverages provide a range of healthier and more affordable options (even in vending machines). e.g., school visits to museums, sports centers, cinemas, and theme parks.	- Lower scores include comments on how to increase the applicability of this measure, typically through regulation by the health authority, although it is recognized that obtaining support for such regulation is challenging, partly due to industry interests and the impact on economic income.<br>- Applicability depends on budgets (low-quality food in the public sector) and responsible technicians.<br>- Challenges include control, surveillance, and enforcement, not assumed by the relevant authorities.<br>- All food procurement policies should be based on restrictions on unhealthy foods.<br>- It has been emphasized that the measure should be implemented for the general population, not just for children. It should focus on sustainable options that prioritize short consumption circuits and establish appropriate monitoring measures.
4	10	When public nutritional education programs are offered, ensure they are scheduled at times that suit people with children (or provide a crèche), fit with diverse working hours and take place in socially acceptable venues (such as community centers) that are accessible locally, either on foot or via public transport.	- In general, this measure is seen as quite feasible.<br>- It is mentioned that the measure would help ensure the success of the intervention, being key to making programs more accessible to the population.<br>- There is no consensus on the importance of parental and caregiver training and involvement. On one hand, active community participation and community centers and educational centers are seen as ideal for this training. On the other hand, it is mentioned that training has little impact in contexts where there is a saturation of advertising messages about unhealthy eating. This would reduce the utility. It is also mentioned that the measure will have varying results depending on income levels (country or family), and it should be considered that "in areas with a high prevalence of food insecurity, these strategies are not very effective because healthy eating depends more on socioeconomic variables than on knowledge and skill-related variables."

Rank	N°	Recommendation	Integration of qualitative information on the perception of applicability
5	2	Promote food-sharing networks, community gardens/greenhouses and farmer's markets to help address food insecurity and improve access to locally grown healthy food.	- This is a measure that highlights the need for strong political will and promotion of public policy ("it can be implemented and executed in some contexts, but strong political will at the local/municipal government level is necessary"). It was also expressed that the promotion of this initiative depends significantly on public policy and legal regulations (for example, on land use modifications, the adaptation or promotion of new spaces in cities, the modification of the type of economic activity in urban centers to agricultural activity, etc.).<br>- It's a recommendation that has sustainability limitations in the medium and long term; therefore, extensive planning is required regarding the supply and demand in cities. A significant number of networks, community gardens, and greenhouses are needed for it to have a real impact.<br>- Access to public spaces for exchanges, markets, and the promotion of urban gardens is a challenge due to the lack of physical space and overcrowding in large urban centers.<br>- The measure exists in some countries (local farmers' markets), but they are limited in terms of time and space.
6	17	Use class time to encourage healthy eating and physical activity. It can be taught as a specific subject (e.g., physical education), as part of other subjects (e.g., science, home economics, mathematics, agriculture), or ideally as a combination of both.	- There isn't a clear consensus among all participants. Various positions have been found: a) Physical education is part of the national education curriculum, so topics related to healthy eating and food preparation should be incorporated into this subject, reinforcing physical education with content about healthy eating and food preparation; b) It should be complemented in natural science and biology classes with nutrition education; c) Evidence suggests that it's better to combine it within the school curriculum (but it doesn't specify how to integrate it), or; d) Incorporate other practical examples of applicability, such as urban/school gardens (spaces where practical techniques and contact with nature, outdoor physical activity, and education take place, which leads to improved eating habits).<br>- Some experts express difficulties in applicability because it's a recommendation that requires significant alignment within the educational regulations. It would involve a time-consuming and planned process. Initially, changes would be made to the curriculum or the introduction of general topics, followed by the transformation into a subject (first technical and regulatory work on the curriculum, followed by teacher training).<br>- It should be part of a comprehensive strategy (policies for healthy school food environments and adequate physical activity, water and sanitation policies in low-income countries, etc.).<br>- It's noted that there is evidence of using simple WhatsApp messages for students and parents, and the strategy should be adapted to different age groups.
7	16	Use posters, verbal prompts, lower (tactical) pricing and product positioning to promote healthier food and drink choices in canteens/bars and vending machines.	- A necessary measure to implement within a framework of health promotion (not to be carried out in isolation).<br>- A measure that already exists in some countries but requires greater effort to implement (related to political and economic aspects).<br>- Posters with messages about healthy foods are important, but they shouldn't compete with unhealthy options in school cafeterias and vending machines. Unhealthy options should not be available in the school environment.<br>- The role of vending machines in the context of environmental sustainability is discussed, and there are doubts about the type of healthy food that such machines could offer.<br>- Consumers don't read labels or posters. There would be a greater impact through social media and graphic expressions.

Rank	N°	Recommendation	Integration of qualitative information on the perception of applicability
8	15	Set up attractive displays of fruits and vegetables in school canteens.	- Difficult to implement without a healthy school food policy that regulates the advertising and sale of ultra-processed products.<br>- It is a feasible and quick recommendation to implement, but it should be part of a comprehensive strategy that complements habit change (nutritional education, incentives to encourage consumption, school garden programs, cooking classes for students, communication campaigns, parental involvement, etc.).<br>- Applicability depends on political will, budget, and efficiency in creating partnerships with local food producers, markets, and sales.
9	6	Authorities should ensure publicly funded venues (e.g., museums, and recreational centers), especially those frequented by children and young people, resist sponsorship or product placement from companies associated with foods and beverages high in fat, sugar, or salt.	- Feasible because as public entities, there is control by the authorities, and it can be extended to transport stations, buses, and parks.<br>- Past experiences have shown the difficulty in applicability due to legal issues related to discrimination against businesses, so "it can be encouraged and promoted, but it's hard to ensure its implementation."<br>- Applicable as long as it is regulated. According to the experience of some participants, the measure requires political commitment, as commercial interests and government favoritism toward some companies can hinder its execution ("some government actors consider sponsorship as an alternative").<br>- Regulations should control advertising (prohibiting marketing harmful products aimed at children) and the sale of junk food in establishments. Therefore, these types of strategies should be applied across all real and virtual spaces.<br>- There are two different opinions on economic aspects: a) it is an option that attracts authorities because it doesn't tie up budgets, and b) it is necessary to consider that some publicly funded facilities do not receive the required budget (from the Central Government), making them seek sponsorship from private companies.
10	14	Introduce school programs that provide fruit and vegetables to pupils during break-time.	- Feasibility depends on political will, economic resources, and the creation of alliances with local producers, sellers, and markets.<br>- It should not be an isolated measure but should be accompanied by an educational component and monitoring and penalties (ultra-processed foods can have higher profits and can be offered clandestinely).<br>- This measure should be part of a national policy but should be linked to policies for purchasing fresh foods from local producers, produced in short, clean, sustainable, family, peasant, seasonal, etc. supply chains.

Rank	N°	Recommendation	Integration of qualitative information on the perception of applicability
11	8	Use welfare benefits and wider schemes to supplement the family food budget and improve eating patterns. e.g., free school meals, free school fruit and vouchers for healthy food outlets.	- It is emphasized that it is debatable whether the strategy is framed as social assistance and not within the framework of the right to food.<br>- School feeding programs must be regulated and have clear regulations to be applicable and consolidated. Furthermore, they could be strengthened by scenarios of social commitment, involving the same producers of ultra-processed foods.<br>- There are programs within the context of malnutrition that could be universalized.<br>- Difficulty in applicability due to lack of initiative and political commitment, as well as the need for financing.<br>- Measures already established in some countries with different experiences and results. Some experiences highlight the importance of reviewing the nutritional quality of school breakfasts and community dining options (concerning sugar, carbohydrates, and fat content).<br>- The option of coupons for exchanging for fresh foods is highlighted as a good measure. Previous experiences (in Chile, Argentina, and Uruguay) demonstrate the importance of regulating targeted spending on healthy food.<br>- Gender-focused labor policies should be promoted to encourage homemade food preparation ("women may take on a double or triple workload both outside and inside the home, opting for unhealthy options to feed their families").
12	21	At workspaces, promote healthier food and drink choices in staff and client restaurants, hospitality suites, vending machines, and shops by using posters, verbal prompts, lower pricing and the positioning of products.	- It should be encompassed within an occupational health policy. It should be regulated in the Labor Code; otherwise, actions will depend on the decisions of the institution and company directors. This could prevent, for example, increasing carbohydrates in food services to boost profits, at the expense of healthy nutrition.<br>- Although it exists, the strategy is not implemented. There must be a strong commitment from institution and company directors to the quality of food for employees.<br>- It should be regulated and integrated into comprehensive plans as part of a global strategy, requiring political will to develop regulations and ensure mandatory compliance.<br>- Positive changes have been mentioned in the post-pandemic period. National menu guidelines for workers should be adopted.
13	18	Use of posters, verbal prompts, and product positioning to promote healthier food and drink choices among the population at food outlets (retailers, restaurants, vending machines, and other food sources in the community).	- It is applicable, but there is an insistence on the need for the measure to be regulated by the state and that it should be reinforced by national-level communication guidelines.<br>- It is entirely achievable, but participants emphasize the difficulty of breaking with the logic of the market. The industry has the power and budget to promote its products.<br>- It is a resource that complements other actions.<br>- There is an insistence that healthy foods should not compete unfairly with unhealthy options in the same sales points, as much as possible. For example, marketing of unhealthy foods and beverages should be restricted first.

Rank	N°	Recommendation	Integration of qualitative information on the perception of applicability
14	1	Ensure local accessibility, either on foot or by public transport, to retailers (supermarkets, corner shops, street markets, and small independent shops) that sell healthy food and drink.	- There is described a great complexity in the applicability of this recommendation due to supply problems, lower profitability, difficulty in distribution, and marketing of healthy products.<br>- The existence of various factors that intervene and influence demand and supply is recognized, describing greater difficulty in preserving and ensuring the biosafety of healthy products due to their shorter shelf life compared to less healthy foods such as ultra-processed foods, which are more readily available and marketable.<br>- The need to strengthen political support or commitment to ensure small-scale production, promotion, sale, and consumption of local and direct healthy foods is emphasized. It is also highlighted that this commitment could ensure the supply in remote areas from production centers and that there are retailers (with healthy products) in areas that may not be of interest to traders.<br>- It is mentioned that the measure could lead to a possible confrontation with large food chains.<br>- Some people express that, more than distance, economic difficulties could be determining access to healthy foods in low-resource contexts.
15	11	If within existing competencies, local authorities should introduce a simple front labelling system for packaged foods, with one single and easy-to-understand label, established independently of the food industry and which guarantees that the food in question is among the healthiest options in its group. Where possible, include in the regulation sanctions for companies that do not comply with the standards.	- There are doubts about the industry's resistance and the labeling model to be applied.<br>- On one hand, it is seen as a positive, useful, and applicable measure, driven by existing legislation or serving to streamline regulations, recommend targeted taxes, and have a significant impact in cities with high overweight and obesity problems. On the other hand, it is seen negatively regarding the confusion that the double measure (in case there is national regulation) could cause for consumers. Several experts recommend that a) the labeling should be regulated by law; b) the measure should be the same for the entire country ("it should be a national, not local measure"); c) the measures should be coordinated (not independent) with the food industry, as it could cause confusion (in consumers) with the manufacturer's label; and d) it is a measure that requires follow-up and dissemination, monitoring, and sanctions for companies that do not meet the standards.<br>- Labeling is a measure that has already been implemented in some countries (although many do not specify whether it is at the local or national level). It is highly promoted in the LAC region, for its impact on reducing high-sugar, high-fat, or high-salt products.<br>- Regarding the industry, the measure may generate more resistance to accepting healthy food ratings.
16	20	At food outlets that prepare recipes include details in menus on the calorie content of meals to help consumers make an informed choice. If the nutritional value of recipes is not known, they should list ingredients and describe the cooking methods used.	- Low applicability in mass food production sites, as well as resistance from large junk food production chains.<br>- High-impact measure on the consumption of unhealthy foods. However, it requires political will and technical support for effective implementation.<br>- It is reflected that nutritional labeling works better.<br>- It is also difficult to apply due to the workload it may represent for individuals (cooks).<br>- As suggestions for improving applicability, it is mentioned: a) the importance of incorporating portion sizes; b) the strategy should also include warning labels on menus for critical nutrients, and c) include detailed nutritional information and ingredients.
17	12	If within existing competencies, local authorities should introduce taxes on sugary drinks and products high in fat and sugar in order to reduce their consumption.	- It needs to be legislated at the national level and involves significant legal complexity. Fiscal measures at the local level could contradict national regulations, making it difficult to implement, control, and monitor locally. It may also face resistance from the food industry.

Rank	N°	Recommendation	Integration of qualitative information on the perception of applicability
			- This measure has already been applied in other contexts, such as beverages and cigarettes, where it has faced applicability and effectiveness challenges. To be effective, it should involve a 20% tax on the product price. It is more challenging to apply to foods.
18	19	Food outlets (retailers, restaurants, vending machines and other food sources in the community) should price healthier foods and beverages at lower costs and use incentives (such as promotional offers) to promote healthier choices.	- There are divergent opinions among participants. It is mentioned that there are previous strategies that have not worked (with basic basket prices), as businesses did not lower their prices even when regulated and encouraged by state tax reductions. On the other hand, the feasibility of the measure is highlighted by providing examples of other successful experiences (publishing prices of healthy foods at market-driven prices or the case of a network of healthy establishments offering discounts to customers).<br>- It is not applicable because healthy foods are more expensive than unhealthy foods. It is emphasized that public policies should be in place to reduce barriers to accessing healthy food, and this measure requires strong support and political will. Regulations or integrated strategies should be used to reduce costs, such as using seasonal fruits, buying from local suppliers, or local governments incentivizing merchants by lowering taxes, which could have a significant impact on reducing fast food and soda consumption.<br>- Reducing costs is challenging, but the use of incentives and coupons may be favorable.<br>- The marketing of unhealthy foods and beverages should be restricted first, and perhaps promotional offers for unhealthy foods and beverages should be discouraged in some way (including taxes).<br>- This measure should be linked to a comprehensive strategy (awareness, education, coordination with retailers and food services).<br>- It is questioned whether government subsidies would fall under the category of incentives.
19	5	Authorities should systematically consider healthier eating options when reviewing applications for new food outlets.	- Participants repeatedly emphasize the importance of regulation (scarcity) for this recommendation to be applicable. "The limitation is the legal regulations, as technical considerations will need to be taken into account for these restrictions," "it is necessary and possible in intrainstitutional policy as public policy", "political will is required," "the guiding and regulatory role of the health authority must be strengthened."<br>- Important measure but low applicability due to: a) the non-healthy food market is an economic priority; b) "bureaucratic requirements [...] to open new food outlets mainly focus on biosafety (microbiological contamination) and do not include a menu and pre-packaged food analysis"; c) a lack of technical capacity to carry out the measure and have rational criteria to promote healthier options (e.g., lack of distribution mapping and control of establishments); d) applicable to formal points of sale, but Latin America and the Caribbean have high informality.<br>- Similar regulations should be monitored and mandatory.

Rank	N°	Recommendation	Integration of qualitative information on the perception of applicability
20	4	Set limits for the number of take-away and junk food outlets in a given area, particularly those near schools.	- There is a high perception of difficulty/complexity in the applicability of the measure, partly due to: a) the high number and distribution of educational centers; b) restrictions on freedom; c) the widespread availability of food outlets; d) the challenge of regulating legal and illegal establishments, informal trade, and unregulated street food vending in many Latin American cities; e) extended hours for workers in the area; g) it is a strategy that needs to be regulated by law, and it requires the commitment and political will to prioritize the interests/rights of the community/consumers over those of businesses ("like many regulatory strategies, approving, implementing, and enforcing it may take time and require strong political will"; "there is no sense of citizenship defending consumer rights, and it will face strong opposition from the industrial sector"); and f) if there is regulation in some countries, there is ambiguity in the regulations, leadership, and a lack of mechanisms and control structures for enforcement.
21	3	Regulate the distance from schools and the opening hours of take-away and other food outlets that specialise in foods high in fat, salt or sugar.	- Several people noted that the measure has a moderate applicability because, in addition to regulation, it requires strong political will and commitment to prohibit/condition the sale and promote healthy spaces.<br>- Some participants mentioned that there is regulation in their countries, but they have encountered various limitations related to applicability, such as:<br>a) Lack of mechanisms and control structures to enforce regulations (there is a conflict of competencies between the central government and municipalities; there is no history of sanctioning nearby establishments despite regulations and municipal ordinances; ambiguity about who should lead the regulation and its steps to follow; limited time for implementation, etc.)<br>b) Complexity in the measure due to regulation and the difficulty in aligning interests between schools and the food industry (difficulty in achieving health outcomes and economic benefits; industry interests can significantly delay a regulation understood as a legally mandatory requirement; difficulty in implementing the measure due to business lobbying).<br>c) The existence of a culture of consuming ultra-processed foods in schools and their surroundings in some regions, leading to a low cultural acceptance of the recommendation. This limitation could question the degree of applicability of the measure, which will depend on the customs of each country.<br>d) Difficult applicability of the measure because in various environments in Latin America and the Caribbean (LAC), the local economy relies on informal work (street or ambulant food sales with extended hours that correspond to the working hours of the area - opening from 5:00 am to 2:00 am).
